# Comprehensive Lifestyle Improvement Program for Prostate Cancer (CLIPP): Protocol for a Feasibility and Exploratory Efficacy Study in Men on Androgen Deprivation Therapy

**DOI:** 10.2196/12579

**Published:** 2019-02-05

**Authors:** Amit Algotar, Chiu-Hsieh Hsu, HH Sherry Chow, Shona Dougherty, Hani M Babiker, David Marrero, Ivo Abraham, Rachit Kumar, Jennifer Ligibel, Kerry S Courneya, Cynthia Thomson

**Affiliations:** 1 University of Arizona Tucson, AZ United States; 2 Dana-Farber Cancer Institute Boston, MA United States; 3 University of Alberta Edmonton, AB Canada

**Keywords:** prostate cancer, lifestyle modification, androgen deprivation therapy, quality of life

## Abstract

**Background:**

Androgen deprivation therapy (ADT) for prostate cancer is associated with adverse cardiometabolic effects such as reduced libido, hot flashes, metabolic syndrome, diabetes, myocardial infarction, and stroke. This reduces quality of life and potentially increases mortality. Several large clinical trials have demonstrated improvements in cardiometabolic risk with comprehensive multimodality lifestyle modification. However, there is a lack of data for such interventions in men on ADT for prostate cancer, and existing studies have used non-standardized interventions or lacked data on metabolic risk factors.

**Objective:**

The Comprehensive Lifestyle Improvement Project for Prostate Cancer (CLIPP) is designed to address these gaps by using an intervention modeled on the Diabetes Prevention Program, a standardized multicomponent intervention with demonstrated effectiveness in reducing cardiometabolic risk factors that has been successfully adapted for multiple disease types including breast cancer.

**Methods:**

A single-arm unblinded clinical trial will be conducted to determine the feasibility of conducting a 24-week comprehensive lifestyle modification intervention that targets weight loss and increased physical activity modeled on the Diabetes Prevention Program in 30 men on ADT for prostate cancer. Secondary aims are to determine the effect of the intervention on cardiometabolic markers and quality of life. The tertiary aim is to determine the effect of the intervention on markers of inflammation and angiogenesis, important mechanisms for prostate cancer progression. Participants will be recruited from the University of Arizona Cancer Center and the surrounding community. The intervention will be delivered weekly in person and over the phone for 16 weeks. For Weeks 16-24, participants receive weekly phone calls from the study health coach to motivate them to continue their lifestyle modification. Questionnaire and biological data are collected at baseline, 12 weeks, and 24 weeks. Body composition using dual-energy x-ray absorptiometry scans will be performed at baseline and end of study.

**Results:**

Based on a sample size of 30, the two-sided 95% confidence interval will not be wider than 0.373 standard deviations for the adherence rate and will not be wider than 0.374 for the retention rate. In addition, the study will have a power of 80% to detect a change of 0.47 standard deviations from baseline for each of the markers investigated in the secondary and tertiary aims assuming a within-subject correlation of 0.20 at a significance level of 5%. The recruitment period is from October 2018 to April 2019.

**Conclusions:**

The aim of CLIPP is to determine the feasibility of conducting a Diabetes Prevention Program–style comprehensive lifestyle modification intervention in men with ADT for prostate cancer and its effects on cardiometabolic adverse effects, quality of life, as well as markers of inflammation and angiogenesis. Results will inform the development of future clinical trials in this population.

**International Registered Report Identifier (IRRID):**

DERR1-10.2196/12579

## Introduction

Androgen deprivation therapy (ADT) has been demonstrated to improve disease-free survival and overall survival in men with prostate cancer (PCa) [1,2]. Androgen deprivation can be accomplished through surgical castration (bilateral orchiectomy) or medical castration through gonadotropin-releasing hormone (GnRH) agonist and antagonist as well as antiandrogen medication [3,4]. Changed hormonal milieu due to ADT is associated with a number of adverse effects such as metabolic syndrome, weight gain, decreased libido, insulin resistance, obesity, sarcopenia, as well as diabetes, myocardial infarction, and cerebrovascular events. These adverse effects not only lower the individual’s quality of life (QoL) but also contribute to increased mortality [3,5,6]. Reports by Cheung and Alibhai demonstrate that ADT is associated with clinically significant decreased QoL especially in the physical and sexual domains [5,6]. In a meta-analysis by Bosco et al, relative risk (RR) for any type of nonfatal cardiovascular disease was 1.38 (95% CI 1.29-1.48) for men with PCa on GnRH agonists, compared with men not treated with ADT [3]. The associations between GnRH agonists and myocardial infarction or stroke were even stronger: RR 1.57 (95% CI 1.26-1.94) and RR 1.51 (95% CI 1.24-1.84), respectively [3].

Comprehensive multicomponent lifestyle modification interventions have demonstrated improvement in metabolic profile and cardiovascular risk factors [7]. The Diabetes Prevention Program (DPP) was a randomized clinical trial (N=3234) comparing the effect of intensive multicomponent lifestyle modification, metformin, or placebo on development of diabetes in persons with impaired glucose tolerance [7]. In this trial, the incidence of diabetes was 11.0, 7.8, and 4.8 cases per 100 person-years for placebo, metformin, and lifestyle modification, respectively. Compared to placebo, incidence of diabetes was reduced by 58% in the lifestyle arm and 31% in the metformin arm. Although there was slight attenuation of the effect, the incidence of diabetes was still lower in the lifestyle arm as compared to the metformin arm (34% and 18%, respectively) after 10 years of follow-up [8]. The 10-year follow-up demonstrated major reductions for blood pressure (systolic, 2-3 mmHg and diastolic, 5-6 mmHg), low-density lipoprotein cholesterol (0.47-0.54 mmol/l), and triglycerides (0.18-0.32 mmol/l) as well as increases in high-density lipoprotein (0.13-0.16 mmol/l) for all the three groups, whereas hyperlipidemia (*P*<.012), hypertension (*P*<.09) [9], and medication use was lower for the lifestyle group. Lack of differentiation between the three groups could potentially be due to all three groups’ receiving the lifestyle intervention when the primary clinical trial ended. The DPP has been successfully adapted for breast cancer patients [10]. In the Lifestyle Exercise And Nutrition (LEAN) study, 100 women with breast cancer were randomized to in-person or phone-based lifestyle modification or usual care. There was a statistically significant decrease in weight (*P*=.004 and *P*=.009 for in-person or phone-based intervention) compared to usual care. Additionally, women who lost >5% weight demonstrated significant improvements in metabolic (insulin and leptin, *P*=.05 and .002, respectively) and inflammatory (C-reactive protein and interleukin-6, *P*=.02 for both) markers [10]. These data served as the premise for the ongoing Breast Cancer Weight Loss trial of a DPP-modified intervention for weight loss in breast cancer survivors [11].

Interventions addressing either exercise or nutrition have demonstrated benefit in men on ADT for PCa [12]. However, there is a lack of data with respect to comprehensive multicomponent standardized lifestyle improvement programs. Recently published results from the Individualized Diet and Exercise Adherence-Pilot (IDEA-P) trial demonstrated that a multicomponent lifestyle modification program is effective in improving mobility performance (*P*<.02), muscular strength (*P*<.01), body fat percentage (*P*<.05), and fat mass (*P*<.03) [13]. However, cardiometabolic risk factors or QoL were not outcome variables in this paper nor was a standardized intervention like the DPP used in the study.

Obesity is an important risk factor for not only cardiometabolic diseases but also for PCa progression. As many as 77% men diagnosed with PCa can be classified as overweight or obese, which is higher than the national average [14]. Obesity has been associated with PCa aggressiveness, progression, and cancer-specific mortality [15]. The Continuous Update Project report on PCa identifies overweight and obesity to be strong risk factors for PCa progression [16]. Hence, an intervention like the DPP with proven efficacy towards reducing weight and improving cardiometabolic risk factors could be hypothesized to have the same effect in men on ADT for PCa.

The current trial addresses the above-mentioned deficiencies by conducting a feasibility and early efficacy study to determine the utility of a DPP-style lifestyle modification intervention on cardiometabolic risk factors and QoL in men on ADT for PCa. Additionally, the effect of the intervention on markers of inflammation and angiogenesis will be determined to understand its impact on PCa progression, as inflammation and angiogenesis are important mechanisms for PCa progression [17-21].

## Methods

### Study Design

The aims of the Comprehensive Lifestyle Improvement Program for Prostate Cancer (CLIPP), a single-arm unblinded clinical trial, are to determine (1) the feasibility of conducting a 24-week comprehensive lifestyle modification intervention in men on ADT for PCa, (2) the effect of a comprehensive lifestyle modification on cardiometabolic risk factors and QoL in men on ADT for PCa, and (3) the effect of a comprehensive lifestyle modification on markers of inflammation and angiogenesis. Regulatory approval for this project has been obtained from the University of Arizona Institutional Review Board and its affiliated hospital system, Banner University Medical Center. Thirty subjects will be recruited from the University of Arizona Cancer Center (UACC), a National Cancer Institute–designated Comprehensive Cancer Center located in Tucson, Arizona, and its surrounding community. The UACC Cancer Survivorship Clinic, Genitourinary Oncology Clinic, and Radiation Oncology Clinic will be involved in recruitment. Flyers will be distributed at various locations around the cancer center informing potential participants about the study. Principal investigators and study staff will reach out to various prostate cancer support groups around the city to recruit participants. Principal investigators and collaborators will introduce the study to potential participants. If the potential participant is willing to participate, their information will be shared with the clinical research associate who will determine eligibility via telephone. Participants recruited through other methods will be instructed to call the clinical research associate directly for timely screening. After eligibility screening, the associate will explain the study purpose, answer questions, and ascertain interest in participation. If the participant agrees, informed consent will be obtained.

### Eligibility

To be eligible for this study, participants are required to meet the following inclusion and exclusion criteria. Since this is a feasibility trial, criteria are purposefully broad. Participants must (1) be men diagnosed with prostate cancer (Stage I-III) within the past 10 years, who are on ADT for their disease, (2) be age 40 or older with at least a 5-year life expectancy, (3) be willing to participate in a lifestyle modification intervention, including all assessments and measurements, (4) speak English, and (5) if participating in any other clinical trial participants, have a 30-day washout period before they can become eligible for this trial. The exclusion criteria for the study consist of the following: (1) PCa survivors currently participating in any other clinical trials, (2) PCa survivors on hospice or with a life expectancy less than 5 years, (3) survivors with Stage IV PCa, (4) having digestive diseases (eg, inflammatory bowel disease, diverticulitis) that make for intolerance of significant increases in plant food intake, and (5) individuals unable to fully comprehend the informed consent or other procedural requirements. Individuals with high adherence to lifestyle guidelines (eg, consuming a vegan diet or participating in moderate to vigorous physical activity for >45 minutes/day, 7 days/week) will be excluded.

### Informed Consent

The investigational nature and objectives of the trial, the procedures, and interventions involved and their attendant risks and discomforts, and potential alternative therapies will be carefully explained to subjects and a signed informed consent obtained. The informed consent form approved by the University of Arizona Institutional Review Board will be used for this. Documentation of informed consent for screening will be maintained in the subject’s research file.

### Study Procedure

Participants will be scheduled for a baseline visit after informed consent has been obtained. This visit will consist of obtaining anthropometric measures, questionnaire data, biological samples, and body composition data using dual-energy absorptiometry (DXA) scan. The intervention will be initiated at this visit. Participants will be provided with a fitness tracking device (FitBit Charge2) that will support the tracking of fitness goals and provides the study team with physical activity data. Participants will keep the fitness tracker after completion of study. Participants will be followed weekly for a duration of 24 weeks. Baseline and every fourth visit will be in-person, whereas the remainder of the intervention support will be provided over the phone. This delivery strategy was adopted based on results of focus group discussions conducted with PCa survivors at the UACC regarding their interests in participating in a lifestyle modification intervention [22]. Anthropometric measurements will be collected at each in-person visit. Questionnaire data and biological samples will be collected at baseline, 12 weeks, and 24 weeks. DXA scans will be obtained at baseline and end of study (24 weeks) to assess body composition changes over time. An exit survey will be carried out at the end of the study to assess acceptance and feasibility including an understanding of their experience and suggestions for improvement. A similar survey will be carried out if the participant decides to terminate their participation earlier than study completion. See Multimedia Appendix 1 for the study timeline.

### Intervention

**Figure 1:**
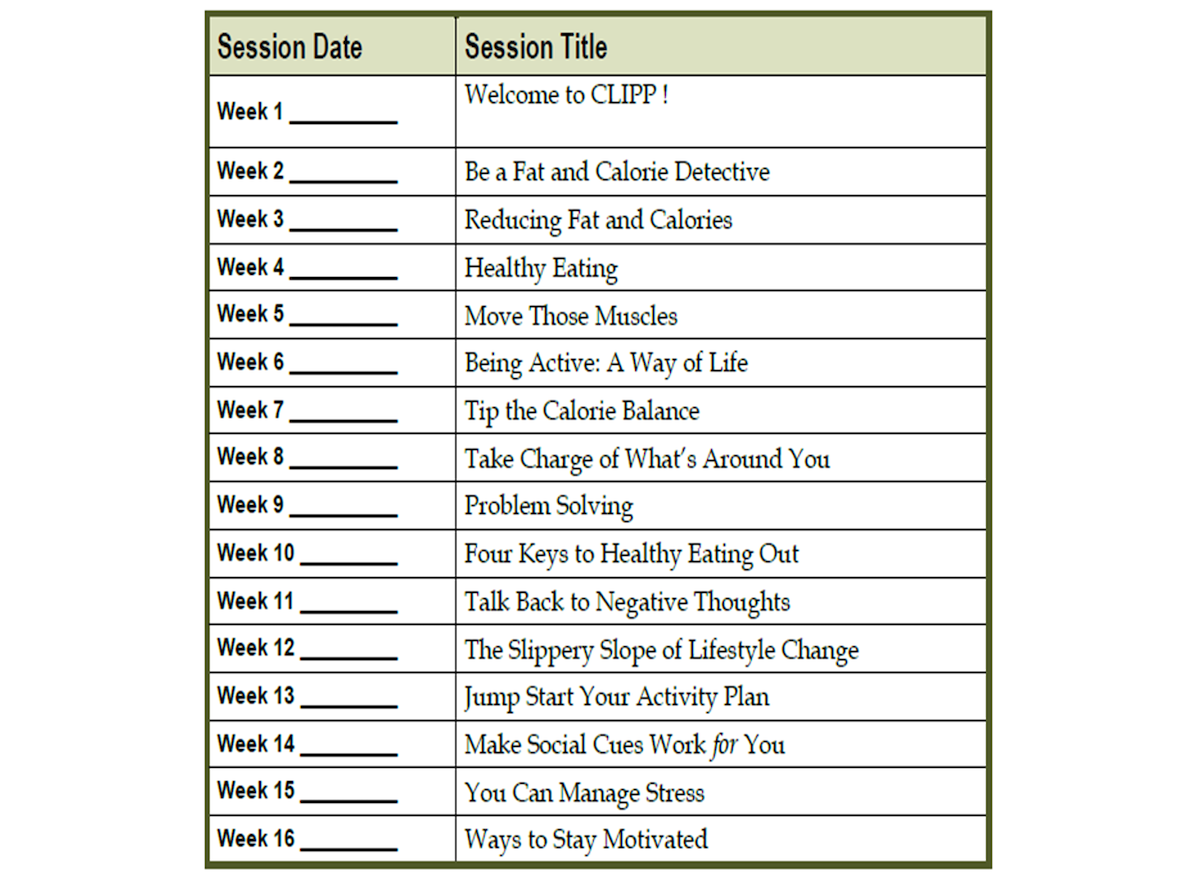
Intervention details.

The intervention has been modeled on the DPP and adapted for men with PCa. The goals of the intervention are to help participants (1) achieve and maintain 7% weight loss from their starting weight (if participant body mass index [BMI] is >25) and (2) achieve and maintain 150 minutes of moderate intensity physical activity weekly. If participant BMI is <25 or if they are already engaging in 150 minutes of moderate intensity physical activity weekly, the goal will be to maintain that for the duration of the trial. The 16-week curriculum covering nutrition, physical activity, as well as supportive strategies to promote and maintain behavior modification was designed to help the participants achieve these goals [7]. Topics covered during the weekly sessions are listed in Figure 1. The curriculum will be taught by an experienced health coach trained by a DPP principal investigator and master trainer. After completion of the curriculum-based intervention, participants will be followed for 8 weeks. During this time, participants will receive weekly calls from the health coach aimed at maintaining motivation and problem solving as well as behavioral goal maintenance. The fundamental components of the intervention are based on social cognitive theory, the theory of planned behavior, and the transtheoretical model [23].

### Outcome Measures

#### Feasibility Measures

Aim 1 of this study is to determine feasibility and hence the outcome measures for this aim will be recruitment, retention, and maintaining protocol adherence by the participants. The recruitment goal is set at 30 participants recruited over a 6-month period. The retention goal is set at 80%, and the adherence goal is set at 75% participation. If 80% (24/30) of participants complete the trial, the retention goal for this trial will be achieved. If participants attend 75% of the intervention delivery visits (in-person and phone combined), they will be considered adherent to the protocol.

#### Efficacy Measures

For Aim 2, anthropometric measures (ie, height in inches, weight in pounds, waist and hip circumference in inches) and blood pressure (ie, systolic and diastolic mm of hg) will be carried out by the same, trained study personnel for all participants at all visits, using established protocols. This will ensure consistency between measurements and reduction in error. Serum samples for metabolic markers (ie, fasting glucose, hemoglobin A1C, lipid panel) will be collected and sent to a certified clinical laboratory. We will determine QoL using standardized and validated questionnaires. The Patient Reported Outcomes Measurement Information System (PROMIS) global physical and mental health scale will be used to determine the overall quality of life, whereas the Expanded Prostate Cancer Index Composite Short Form (EPIC-26) will be used to assess disease-specific QoL. Preliminary efficacy will be established if the intervention results in a significant decrease in body weight, body fat, and metabolic indices as well as improvement in general and disease-specific QoL over the course of the trial (24 weeks). For Aim 3, serum collected at baseline, 12 weeks, and 24 weeks will be processed for markers of inflammation (ie, Interleukin-6, Interleukin 1-beta, Interleukin-8, stromal cell derived factor 1-alpha, and basic fibroblast growth factor) and angiogenesis (ie, vascular endothelial growth factor and plasma placental growth factor) using enzyme linked immunosorbent assay (ELISA). Kits will be purchased from Meso Scale Discovery (Rockville, MD) and R&D Systems (Minneapolis, MN). Prior literature demonstrates changes in these markers due to ADT [4], hence, the reason for choosing these markers.

#### Ancillary Measures

In addition to the outcome measures mentioned above, ancillary measures will also be collected that will be used for correlative analyses in the future. Other ancillary measures will consist of Pittsburgh Sleep Quality Index to assess sleep quality, Arizona Food Frequency Questionnaire to assess food patterns, DXA scans for body composition assessment, and urine samples. Questionnaires and urine samples will be collected at baseline, 12 weeks, and 24 weeks and stored for future analyses. DXA scans will be conducted at baseline and end of study. Extra serum samples will also be stored in -80°Celsius freezers for future use. The samples will be housed in the central freezer facility of the University of Arizona Cancer Center, which is monitored through a 24-hour alarm system.

### Statistical Considerations

#### Statistical Analysis

The primary aim of this study is to determine feasibility of conducting a lifestyle modification intervention in a population of men with prostate cancer on ADT. This will be determined by calculating the study initiation rate, retention rate, and adherence rate. The study initiation rate will be calculated by dividing the total number of subjects that passed screening by the number started in the study after appropriate informed consent procedures. Retention rate will be calculated by dividing the total number of participants initiated by total number of participants in the study at 12 and 24 weeks. Retention goal for this trial is 80%, and hence participation 24 subjects in the trial at 12 and 24 weeks will satisfy this goal. The adherence rate hypothesized for this study is 75%. Participants will be determined to be adherent if they attend 75% intervention sessions (in-person and telephone intervention sessions combined). The overall adherence and retention rates and the associated 95% confidence intervals will be reported at 12 and 24 weeks. The secondary aim of this project to detect the effect of lifestyle modification on cardiometabolic risk factors and QoL. The tertiary aim of this project is to determine the effect of lifestyle modification on markers of inflammation and angiogenesis. Baseline characteristics will be described using mean and standard deviation for continuous variables and frequency and the associated proportions for categorical variables. Each outcome, for the secondary and tertiary aims, will be measured at 3 time points (ie, baseline, 12 weeks, and 24 weeks). For each outcome, two-sided 95% confidence intervals will be constructed for changes from baseline at both 12 and 24 weeks. In addition, a linear mixed-effects models will be fitted to explore the trajectory of changes overtime. If necessary, baseline value of the marker of interest (eg, cardiometabolic markers and QoL for secondary aim and inflammatory and angiogenic markers for the tertiary aim), age, race, serum prostate-specific antigen (PSA), and Gleason score will be adjusted for in the mixed-effects models. The changes in each of the outcomes over time will allow us to evaluate whether lifestyle modification can improve cardiometabolic risk and QoL (secondary aim) as well as inflammation and angiogenesis (tertiary aim). Analysis will be carried out using intent-to-treat (all participants) and modified intent-to-treat (restricting the analyses to participants who were adherent) approaches in order to determine if study adherence plays a role in mediating the relationship between lifestyle modification and outcome variables. Exit surveys will be carried out at the end of the study or if the participant decides to drop out of the study early. These qualitative and quantitate surveys will help understand participant satisfaction with the intervention and its delivery modalities, which will be helpful in planning the next phases of this project.

#### Sample Size and Power

Based on a sample size of 30, the two-sided 95% confidence interval will not be wider than 0.373 standard deviations for the adherence rate and will not be wider than 0.374 for the retention rate at each follow-up visit. In addition, it will have a power of 80% to detect a change of 0.47 standard deviations from baseline over two follow-up visits for each of the markers investigated in the secondary and tertiary aims, assuming a within-subject correlation of 0.20 at a significance level of 5%.

## Results

Results from this arm open label clinical trial will allow us to determine the feasibility of conducting a DPP based intervention in men on ADT for PCa. Results are expected to be available by October 2019 and will inform the development of future trials in this population.

## Discussion

### Principal Considerations

The Comprehensive Lifestyle Improvement Project for Prostate Cancer (CLIPP) is designed to investigate the feasibility and early efficacy of a comprehensive multimodality intervention modeled after the DPP on cardiometabolic risk factors and QoL, as well as markers associated with inflammation and angiogenesis in men treated with androgen deprivation therapy for prostate cancer. This will be the first study in literature to investigate the utility of a DPP-based intervention on cardiometabolic risk factors and pathways associated with tumor progression in men with PCa on ADT. Although ADT improves disease-free survival and overall survival in men with PCa [1,2], its adverse effects lower the individual’s QoL and contribute to higher mortality. If an effective intervention is identified to mitigate the adverse effects associated with ADT, it could potentially improve the individual’s QoL as well as reduce their risk of treatment-related mortality. Results from the CLIPP study have the potential to provide data on the effect of the lifestyle modification intervention on QoL, cardiometabolic risk factors, as well as markers of inflammation and angiogenesis. Results for the primary aim are expected to be available by October 2019.

### Strengths and Limitations

The strengths of this study lie in its application of an evidence-based and effective multicomponent lifestyle modification intervention. This intervention has been successfully adapted for other disease types including breast cancer with favorable results. The standardized intervention with proven success in multiple disease settings, including cancer, sets this study apart from other comparable studies in current literature. The limitations of this study include its non-randomized treatment assignment and lack of a control group. Since this is a feasibility study, this is an accepted study design and a randomized study with an appropriate control arm would be the next step based on the study results.

### Conclusion

Findings from this study will address a critical gap in current literature by providing data regarding the feasibility of conducting a multimodality lifestyle modification intervention in men on ADT for prostate cancer and its impact on cardiometabolic risk factors, quality of life, as well as markers associated with inflammation and angiogenesis. These data are critical in developing future clinical trials in this group of patients. Additionally, the findings hold potential to open new avenues of research such as the impact of lifestyle modification on prostate cancer progression.

## Appendix

Multimedia Appendix 1 Study timeline.

Multimedia Appendix 2 Peer-review report.

Multimedia Appendix 3 Peer-review report.

## Abbreviations

ADT: androgen deprivation therapy
CLIPP: Comprehensive Lifestyle Improvement Program for Prostate Cancer
DPP: Diabetes Prevention Program
ELISA: enzyme linked immunosorbent assay
EPIC-26: Expanded Prostate Cancer Index Composite-Short Form
GnRH: gonadotropin-releasing hormone
IDEA-P: Individualized Diet and Exercise Adherence-Pilot
LEAN: Lifestyle Exercise And Nutrition study
PCa: prostate cancer
PROMIS: Patient Reported Outcomes Measurement Information System
QoL: quality of life
RR: relative risk
UACC: University of Arizona Cancer Center
