# Supplementary material for: Comprehensive Lifestyle Improvement Program for Prostate Cancer (CLIPP): Protocol for a Feasibility and Exploratory Efficacy Study in Men on Androgen Deprivation Therapy
Source: JMIR Res Protoc. 2019 Feb 5;8(2):e12579. doi: 10.2196/12579 (PMC6379812; doi:10.2196/12579)
Supplement: Multimedia Appendix 1 [file resprot_v8i2e12579_app1.docx]

| **Table 1:** |  | Intervention | | | | | | | | | | | | | | | | Follow-up | | | | | | | |
| --- | --- | --- | --- | --- | --- | --- | --- | --- | --- | --- | --- | --- | --- | --- | --- | --- | --- | --- | --- | --- | --- | --- | --- | --- | --- |
| Week | -1 | 1 | 2 | 3 | 4 | 5 | 6 | 7 | 8 | 9 | 10 | 11 | 12 | 13 | 14 | 15 | 16 | 17 | 18 | 19 | 20 | 21 | 22 | 23 | 24 |
| Screening | X |  |  |  |  |  |  |  |  |  |  |  |  |  |  |  |  |  |  |  |  |  |  |  |  |
| Informed Consent | X |  |  |  |  |  |  |  |  |  |  |  |  |  |  |  |  |  |  |  |  |  |  |  |  |
| Anthropometric measurements |  | X |  |  | X |  |  |  | X |  |  |  | X |  |  |  | X |  |  |  |  |  |  |  | X |
| Self-reported weight |  |  | X | X |  | X | X | X |  | X | X | X |  | X | X | X |  | X | X | X | X | X | X | X |  |
| Physical activity* |  |  | X | X | X | X | X | X | X | X | X | X | X | X | X | X | X | X | X | X | X | X | X | X |  |
| Questionnaires |  | X |  |  |  |  |  |  |  |  |  |  | X |  |  |  |  |  |  |  |  |  |  |  | X |
| Blood collection |  | X |  |  |  |  |  |  |  |  |  |  | X |  |  |  |  |  |  |  |  |  |  |  | X |
| Intervention (In-person) |  | X |  |  | X |  |  |  | X |  |  |  | X |  |  |  | X |  |  |  |  |  |  |  |  |
| Intervention (Telephone) |  |  | X | X |  | X | X | X |  | X | X | X |  | X | X | X |  |  |  |  |  |  |  |  |  |
| Motivational phone calls |  |  |  |  |  |  |  |  |  |  |  |  |  |  |  |  |  | X | X | X | X | X | X | X |  |
| DXA scan |  | X |  |  |  |  |  |  |  |  |  |  |  |  |  |  |  |  |  |  |  |  |  |  | X |

*Physical activity tracking will be carried out by accessing participant’s FitBit account weekly.
