# Supplementary material for: Comprehensive Lifestyle Improvement Program for Prostate Cancer (CLIPP): Protocol for a Feasibility and Exploratory Efficacy Study in Men on Androgen Deprivation Therapy
Source: JMIR Res Protoc. 2019 Feb 5;8(2):e12579. doi: 10.2196/12579 (PMC6379812; doi:10.2196/12579)
Supplement: Multimedia Appendix 2 [file resprot_v8i2e12579_app2.pdf]

UACC Scientific Review Committee
1515 N Campbell Avenue
PO Box 245024, Room 1955
Tucson AZ 85724-5024

THE UNIVERSITY OF ARIZONA. CANCER CENTER

Phone: 520- 626-1155
Fax: 520-626-9001
lgarland@uacc.arizona.edu
UACC-SRC@uacc.arizona.edu

UACC SCIENTIFIC REVIEW COMMITTEE
OUTCOME REPORT

Protocol title: Comprehensive Lifestyle Improvement Program for Prostate Cancer Survivors

Protocol number: 29472

Principal investigator: Amit Algotar, MD

Sponsor: Arizona Cancer Center

Date: 4/27/2018

Statistical review (i.e., approval, pending, etc.): pending

Pharmaceutical review (i.e., approval, pending, etc.): n/a

Findings/determination: Defer with full committee review

Comments: This study was reviewed by the Scientific Review Committee for scientific merit, study design and feasibility. During the review, concerns were raised that require a response.

1. It is not clear that this submission to the SRC is to cover the entirety of the project (clinical procedures of the study as well as biomarker studies) or the biomarker studies only. Is this project adapting a pre-existing protocol or is this an entirely new protocol? Please clarify this by providing a protocol or protocols that delineate the entirety of the study including Background and Rationale, Study Procedures, Methods, Eligibility, Biomarkers, Statistical Plan, Monitoring, Study Calendar. The currently submitted F200 does not provide sufficient information for comprehensive review of this project.

2. Please contact the UACC Biostatistics Shared Resources to develop a statistical plan for the study.

3. What is the justification for choosing a sample size of 30 patients for enrollment?

4. Clarify the following inclusion/exclusion criteria: Can participants currently be on active treatment beyond ADT alone or have been treated with chemotherapy and/or radiation in past? Are Stage IV patients able to be included?

5. Who will screen potential participants for robustness required to fulfill the LM modification interventions and using what measures?

UACC Scientific Review Committee
1515 N Campbell Avenue
PO Box 245024, Room 1955
Tucson AZ 85724-5024

THE UNIVERSITY OF ARIZONA.
CANCER CENTER

Phone: 520- 626-1155
Fax: 520-626-9001
lgarland@uacc.arizona.edu
UACC-SRC@uacc.arizona.edu

6. What is the rationale for the eligibility criterion of ages 40 years or older?

7. Clarify the total duration of the study. The IRB application states that the LM intervention will be over 24 weeks, but also, "Total duration of the intervention will be 16 years with total follow up of 6 months".

8. How much blood is to be drawn from each participant over the life of the study? Where will these samples be stored?

9. Please provide the funding source(s) for this study.

Submit a written response to the Committee's concerns and any other supporting or revised documents into Oncore.

Sincerely,

Linda Garland, MD
Chair, UACC Scientific Review Committee
