# Supplementary material for: Comprehensive Lifestyle Improvement Program for Prostate Cancer (CLIPP): Protocol for a Feasibility and Exploratory Efficacy Study in Men on Androgen Deprivation Therapy
Source: JMIR Res Protoc. 2019 Feb 5;8(2):e12579. doi: 10.2196/12579 (PMC6379812; doi:10.2196/12579)
Supplement: Multimedia Appendix 3 [file resprot_v8i2e12579_app3.pdf]

UACC Scientific Review Committee
1515 N Campbell Avenue
PO Box 245024, Room 1955
Tucson AZ 85724-5024

THE UNIVERSITY OF ARIZONA. CANCER CENTER

Phone: 520- 626-1155
Fax: 520-626-9001
lgarland@uacc.arizona.edu
UACC-SRC@uacc.arizona.edu

**UACC SCIENTIFIC REVIEW COMMITTEE**
**OUTCOME REPORT**

**Protocol title:** Comprehensive Lifestyle Improvement Program for Prostate Cancer Survivors

**Protocol number: 29472**

**Principal investigator**: Amit Algotar, MD

**Sponsor:** Arizona Cancer Center

**Date:** 6/4/2018

**Statistical review (i.e., approval, pending, etc.):** approved

**Pharmaceutical review (i.e., approval, pending, etc.):** n/a

**Findings/determination: APPROVED**

**Comments:** This study and the PI's response dated, 5/4/2018, were reviewed by the Scientific Review Committee on 5/11/2018. The response is appropriate and addresses the committee's concerns.

Sincerely,

Linda Garland, MD
Chair, UACC Scientific Review Committee
